# Bronchoalveolar-Lavage-Derived Fibroblast Cell Line (B-LSDM7) as a New Protocol for Investigating the Mechanisms of Idiopathic Pulmonary Fibrosis

**DOI:** 10.3390/cells11091441

**Published:** 2022-04-24

**Authors:** Laura Bergantini, Miriana d’Alessandro, Sara Gangi, Dalila Cavallaro, Giuseppe Campiani, Stefania Butini, Claudia Landi, Luca Bini, Paolo Cameli, Elena Bargagli

**Affiliations:** 1 Respiratory Disease and Lung Transplant Unit, Department of Medical Sciences, Surgery and Neuroscience, Siena University, 53100 Siena, Italy; dalessandro.miriana@gmail.com (M.d.); sara.gangi@student.unisi.it (S.G.); cavallarodalila@gmail.com (D.C.); paolocameli88@gmail.com (P.C.); bargagli2@gmail.com (E.B.); 2 Department of Biotechnology, Chemistry and Pharmacy, DoE Department of Excellence 2018–2022, University of Siena, Via Aldo Moro 2, 53100 Siena, Italy; giuseppe.campiani@unisi.it (G.C.); butini3@unisi.it (S.B.); 3 Department of Life Sciences, University of Siena, 53100 Siena, Italy; landi35@unisi.it (C.L.); luca.bini@unisi.it (L.B.)

**Keywords:** BAL, fibroblast, IPF

## Abstract

Background: The use of BAL to study ILDs has improved our understanding of IPF pathogenesis. BAL fluid is routinely collected and can be considered a clinical and research tool. The procedure is well tolerated and minimally invasive. No specific cell lines from BAL or immortalized cell lines from IPF patients are available commercially. A method to quickly isolate and characterize fibroblasts from BAL is an unmet research need. Materials and methods: Here we describe a new protocol by which we isolated a cell line from IPF. The cell line was expanded in vitro and characterized phenotypically, morphologically and functionally. Results: This culture showed highly filamentous cells with an evident central nucleus. From the phenotypic point of view, this cell line displays fibroblast/myofibroblast-like features including expression of alpha-SMA, vimentin, collagen type-1 and fibronectin. The results showed high expression of ROS in these cells. Oxidative stress invariably promotes extracellular matrix expression in lung diseases directly or through over-production of pro-fibrotic growth factors. Conclusions: Our protocol makes it possible to obtain fibroblasts BAL that is a routine non-invasive method that offers the possibility of having a large sample of patients. Standardized culture methods are important for a reliable model for testing molecules and eventual novel development therapeutic targets.

## 1. Introduction

Progressive fibrosing interstitial lung diseases (ILDs) are associated with high mortality and morbidity. [1] Idiopathic pulmonary fibrosis (IPF) is a chronic, irreversible, progressive ILD of unknown etiology associated with poor prognosis and a mean survival of about 5–6 years from the onset of symptoms [2,3]. It is difficult to diagnose since it has clinical and radiological signs similar to those of other fibrotic ILDs [4]. Risk factors for IPF include smoking, genetic predisposition and environmental variables linked to pathological alteration of lung epithelium [5]. The exact pathogenetic mechanisms leading to the development of IPF are not fully understood [6]. Fibroblast cultures are useful for the study of IPF, since these cells are fundamental in the pathogenesis of this rare lung disease [7].

Release of fibrotic mediators induces deposition of pathological matrix by activated fibroblasts. Fibroblasts are activated in a pro-fibrotic environment, where they lose their epithelial phenotype and acquire a mesenchymal phenotype [8]. Many molecular factors, such as high levels of transforming growth factor-beta (TGF-β) that induce epithelial–mesenchymal transition, and expression of α-smooth muscle actin (α-SMA) that actively produces extracellular matrix [9,10], may be involved. There is consequently an additional differentiation of fibroblast to myofibroblast, followed by deposition of matrix [11]. The presence of fibroblasts is an evident sign of fibrotic processes, and has been an important starting point for a number of studies [12].

The use of broncho alveolar lavage (BAL) to study ILDs has improved our understanding of their pathogenesis. BAL fluid is routinely collected and can be considered a clinical and research tool. The procedure is well tolerated and minimally invasive, posing little risk for patients [13,14]. Several studies have evaluated BAL in IPF using immunochemistry, proteomics and real time polymerase chain reaction (RT-PCR) or RNA microarrays to assess the production of cytokines and chemokines with pro-inflammatory or pro-repair/fibrogenic activities [15,16,17]. A mouse model of bleomycin-induced fibrosis failed to represent fibrotic processes in the human lung, some aspects and molecular mechanisms being quite different [18]. The availability of cell lines from tissue is also limited, often being obtained from explanted lungs with end-stage disease. No specific cell lines from BAL or immortalized cell lines from IPF patients are available commercially [19]. A method to quickly isolate and characterize fibroblasts from BAL is an unmet research need. Here we describe a new protocol by which we isolated a cell line from an IPF patient. The cell line was expanded in vitro and characterized phenotypically, morphologically and functionally.

## 2. Materials and Methods

### 2.1. Patient Characteristics

The present study was approved by the Regional Ethics Committee (“Epigenetic and proteomic approaches to innovative targeted therapies for idiopathic pulmonary fibrosis” Protocol code: HIDE-IPF 01, Protocol number: 18545) and was conducted according to current national and international guidelines [2]. Samples were anonymized prior to cell processing. Diagnosis of IPF was based on international criteria, including multidisciplinary discussion in the Interstitial Lung Disease Group and gathering informed consent to explicit use of biological samples and any derivatives for research purposes. The patient underwent bronchoscopy with BAL for diagnostic purposes. Aliquots of BAL fluid not used for diagnosis were processed for additional research procedures.

### 2.2. Collection and Processing of BAL

Bronchoscopy with bronchoalveolar lavage was performed for diagnostic purposes in line with ATS/ERS Guidelines on BAL [20]. BAL fluid was obtained by instillation of four 60-mL aliquots of saline solution by fibrobronchoscope (Olympus IT-10) wedged in a subsegmental bronchus of the middle lobe or lingula. The first sample was kept separate from the others and was not used for immunological tests. BAL was filtered through sterile gauze. Cellularity was determined by cytocentrifuging glass slides for 5 min at 40× *g*. Staining was performed with a May Grunwald Giemsa stain kit (DiaPath, Italy, Europe). At least 500 cells were counted. Cell viability was determined by trypan blue exclusion in a Burker chamber.

### 2.3. Isolation of Adhering Cells

Human lung fibroblasts were isolated from BAL fluid of a 65-year-old male patient with IPF. The sample was filtered with 300 µm sterile gauze (Biosigma S.p.A. a Dominique Dutscher Company, Italy, Europe). The pellet was treated with 0.91 U/mg Dyspase (Gibco, ThermoFisher Scientific, Waltham, MA, USA) for 90 min to separate cells grown in vitro; the enzyme is very mild and does not damage cell membranes. It was also used to prevent clumping in culture. Then the cells were seeded in a 12-well plate at a concentration of 500,000/mL for 24 h with RPMI supplemented with 10% fetal bovine serum (FBS) (Gibco, ThermoFisher Scientific, Waltham, MA, USA) and 0.5% penicillin-streptomycin (Merck KGaA, Darmstadt, Germany). After 24 h, the supernatant was removed with non-adhering cells, which were washed with phosphate-buffered saline (PBS), adding 0.5 mL of Fibroblast Growth Basal Medium (Lonza, NC, USA) supplemented with 0.050 mL 1X insulin, 1X human fibroblast growth factor-2 (FGF-2) and 0.50 mL FBS, and 0.50 mL 1X Gentamycin1000 (all purchased from Lonza, NC, USA). The complete medium was changed once a week or when appropriate. Cells were maintained in 5% CO_2_ at 37 °C.

### 2.4. Fibroblast-Like Cell Culture Derived from BAL

After 21 days, a good number of spindle-shaped cells were attached to the bottom of the wells. The cells were divided, and the culture was continued to confluence, half with complete medium and half with RPMI complete medium.

1X Trypsin-EDTA treatment (Euroclone S.p.A., Milan, Italy) was used to detach the cells. Briefly, 150 µL/mL of 1X Trypsin-EDTA was added to the well plate and incubated for 5 min at 37 °C. The action of trypsin was blocked with RPMI basal medium. With the help of a cell scraper, cells were detached from the bottom of the plate. The pellets obtained were collected and further expanded in 6-well plates or in 25-cm^2^ flasks as appropriate. Aliquots of these cells were then assessed for expression of markers specific for fibroblast/myofibroblast differentiation using specific moAbs. They then underwent cyto-fluorographic analysis, as described below. Fibroblasts were stored in liquid nitrogen at −196 °C.

### 2.5. Fibroblast Cell Culture

Human lung fibroblasts (HLF) from IPF donor (DHLF-IPF—Diseased Human Lung Fibroblasts, Idiopathic Pulmonary Fibrosis) were purchased from Lonza Walkersville Inc. (Lonza Inc., Walkersville, MD, USA) and cultured in complete FMB (Lonza Inc., Walkersville, MD, USA). Cryopreserved ampule of Diseased Human Lung Fibroblasts, Idiopathic Pulmonary Fibrosis (DHLF-IPF) containing ≥ 500,000 cells was purchased and expanded until the sufficient number of cells was achieved. Cells from passages 6 to 11 were used in all experimental studies.

### 2.6. Characterization of Phenotype

To phenotype the cells, five different fluorochrome-conjugated monoclonal antibodies were used: mouse anti-human CD105 V450 (BD Bioscience, San Jose, CA, USA), fixable viability dye eFluor™ 506 (Invitrogen, Carlsbad, CA, USA), anti-collagen type I FITC polyclonal antibody (ThermoFisher Scientific, Waltham, MA, USA), Alexa Fluor 647 anti-mouse fibronectin (BD Bioscience, San Jose, CA, USA), anti-human vimentin monoclonal antibody PE (ThermoFisher Scientific, Waltham, MA, USA), anti-human alpha-smooth muscle actin PerCP-conjugated antibody (R&D Systems, Bio-Techne, Minneapolis, USA). The appropriate amount of each moAb was according to the manufacturers’ instructions. Briefly, 10^6^ cells/mL were stained with the appropriate amount of mouse anti-human CD105 V450 and fixable viability dye eFluor™ 506 followed by 30 min incubation at 4 °C. Then the cells were washed with sufficient buffer (FBS inactivated by 30 min at 56 °C in a thermostatic bath). Cells were thoroughly resuspended in 250 μL BD Cytofix/Cytoperm solution per tube and incubated for 20 min at 4 °C. Cells were washed twice in a buffer containing a cell permeabilizing agent (BD Perm/Wash™ buffer, Cat. 554723). Fixed/permeabilized cells were thoroughly resuspended in 50 μL BD Perm/Wash buffer containing a predetermined optimal concentration of anti-collagen type I FITC, anti-fibronectin Alexa Fluor 647, anti-human vimentin PE and anti-human alpha-smooth muscle actin PerCP-conjugated antibody. They were incubated at 4 °C for 30 min in the dark and washed twice with BD Perm/Wash buffer and resuspended in staining buffer prior to flow cytometric analysis. Cytofluorimetric analysis was then performed with a FacsCanto II cytofluorimeter (Becton Dickinson, NJ, USA).

### 2.7. Wound Scratch Test

Fibroblasts were seeded in 12-well plates to a density of 10^5^/mL, and allowed to adhere and proliferate to semi-confluence in complete medium. Two wells were stimulated with TGF-β1 (Sigma Aldrich, St Louis, MO, USA) at a concentration of 2 µg/mL. A full-diameter scratch was then made on the cell layer in each culture well, using the tip of a sterile 200-μL micropipette. Photographs were taken through an inverted microscope at 0, 24 and 48 h. The number of cells encompassed by the lesion area at the three times was used as a parameter to define rate of growth conditions under the experimental settings assessed. Images of cells filling the gaps were acquired 0, 24 and 48 h after generating the lesion. The original boundaries of each lesion area were superimposed on the frames captured from the same plate in the same position at each experimental timepoint; cells within boundaries were then counted using the National Institute of Health ImageJ free software 1.48v.

### 2.8. Cell Sorting with Anti-Fibroblast Microbeads

Cell sorting with microbeads was conducted in solution containing 50 mL PBS and 250 ng bovine serum albumin. The cells were centrifuged at 300× *g* for 10 min. The cell pellet was resuspended in 80 μL solution, previously prepared for 10^7^ cells, with addition of 20 μL anti-fibroblast microbeads, incubated for 30 min in the dark at room temperature. Then the cells were washed by adding 1 mL buffer and centrifuged at 300× *g* for 10 min. After removing the supernatant, the cells were resuspended in 500 μL buffer. Magnetic separation began with 30 μm nylon mesh (Pre-Separation Filters, 30 μm, # 130-041-407) to remove cell clumps that could clog the column. The column was placed in the magnetic field of a suitable MACS Separator and rinsed with 500 μL buffer. Flow-through containing unlabeled cells was collected. This passage was repeated three times. The column was removed from the separator and placed on a suitable collection tube. The magnetically labelled cells were immediately flushed out by firmly pushing the plunger into the column.

### 2.9. Detection of Oxidative Stress

When cells are stressed, they produce reactive oxygen species. To investigate the general oxidative state of fibroblasts, we used CellRox Green Flow Cytometry Assay (ThermoFisher Scientific, Waltham, MA, USA). Positive and negative controls were added to the assay, namely tert-butyl hydroperoxide (TBHP) oxidant and N-acetylcysteine (NAC) antioxidant, respectively. A 50 mM intermediate dilution of TBHP was reconstituted with PBS, while 10 mg NAC was reconstituted with 245 μL PBS to make a 250 mM solution.

The positive control was obtained by adding 4 μL TBHP and 496 μL PBS. To obtain the negative control, 10 μL NAC and 490 μL PBS were added to the well and incubated for 1 h under normal growth conditions. After 1-h incubation with NAC, 4 μL TBHP was added to the negative control cells and incubated for 45 min. A mix composed of 1 μL Cell ROX reagent and 9 μL DMSO was added to the well with the negative control and to our cell culture. During the final 15 min of staining, 1 μL 5 μM SYTOX Red Dead Cell stain solution was added to 1 μL DMSO. Then the cells were detached with trypsin. The samples were analyzed immediately by flow cytometry using 488-nm excitation for Cell ROX Green and 639-nm excitation for SYTOX red stain.

**Figure 1:**
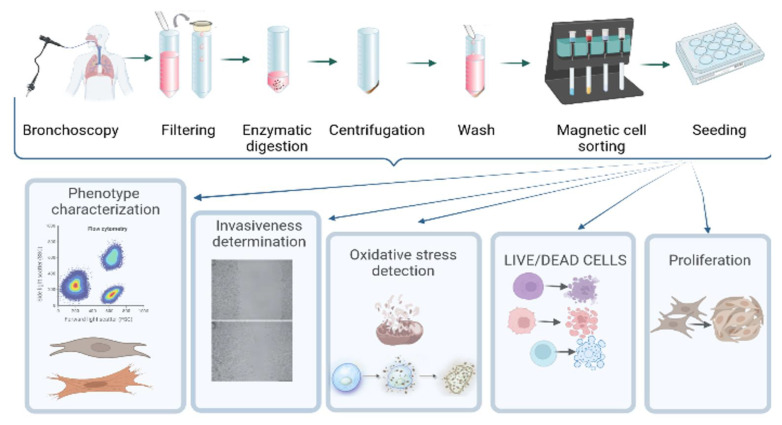
Flowchart of the study.

The flowchart of the experiment is reported in Figure 1.

### 2.10. Statistical Analysis

Where appropriate, the Mann–Whitney test was used to determine the significance of *p*-values: *p* < 0.05 (*) *p* < 0.01 (**), *p* < 0.01 (***), *p* < 0.001 (****). The analysis was performed through Kaluza software (Beckman Coulter, Inc., Brea, CA, USA) and GraphPad 9.0 (GraphPad Software, Inc., San Diego, CA, USA). Flowchart and graphical abstract were created with Biorender.com, accessed on 29 March 2022.

## 3. Results

### 3.1. Patient’s Clinical and Diagnostic Features

The patient used to develop the fibroblast cell line was an ex-smoker (67 years old; male) who presented at the Respiratory Diseases Unit of Siena University Hospital with cough and dyspnea. A diagnosis of IPF was performed according to Official ATS/ERS/JRS/ALAT Clinical Practice Guidelines [2]. HRCT of the lungs showed UIP pattern with ground glass opacities. Lung function testing showed restrictive deficit. BAL was obtained from the middle lobe for diagnostic purposes after multidisciplinary discussion. Cytofluorimetric analysis of BAL showed 91% alveolar macrophages and less than 5% lymphocytes.

### 3.2. Establishment of a Morphologically Unique Fibroblast Cell Line (B-LSDM7) from IPF

**Figure 2:**
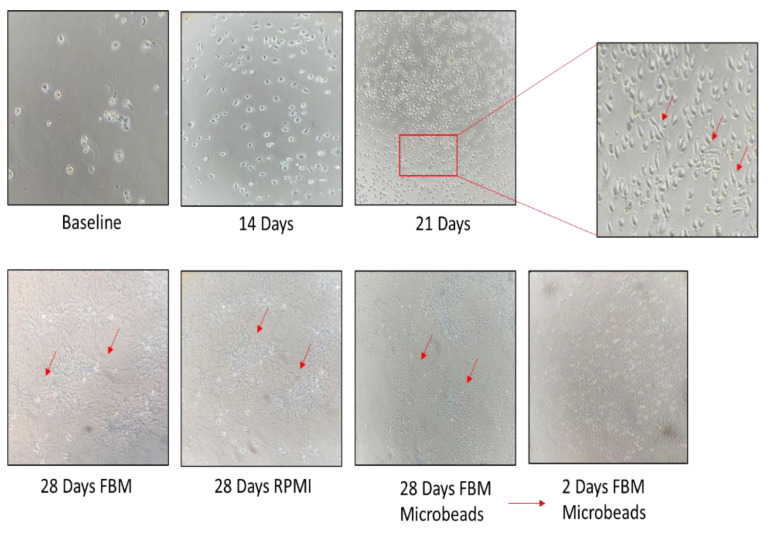
The inverted microscope were used for photo at time 0, 14, 21 and 28 days of cell cultures in both, RPMI and FBM experiments. At any timepoint, the number of cells were counted.

**Figure 3:**
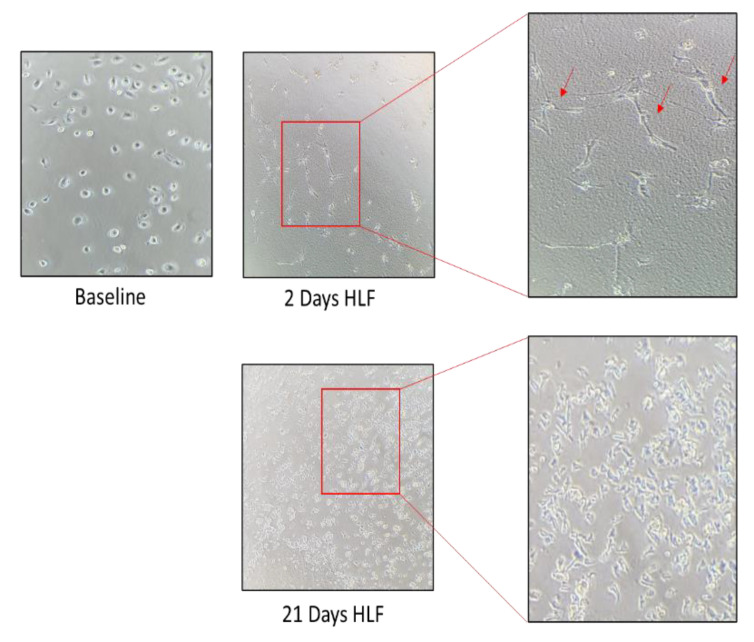
The inverted microscope were used for photo at time 0, 2, and 21 days of HLF cell cultures At any timepoint, the number of cells were counted. Spindle shaped cells was observed after two days of seeding.

We characterized a fibroblast cell line derived from BAL fluid of the IPF patient. The cell line was expanded until fibroblasts started to grow and form colonies (about 3 weeks) according to the protocol reported above. These cells were continuously expanded for 21 days, when they were split into three flasks: the first grown with enriched FBM, the second grown with RPMI, 10% FBS and 5% penicillin/streptomycin, and the third sorted using anti-fibroblast microbeads. Cultured B-LSDM7 cells were observed by inverted light microscope. At 28 days, some of these cells were phenotyped and the rest were frozen in liquid nitrogen. At each passage, cells were counted by trypan blue exclusion in a Burker chamber. As shown in Figure 2, the number of B-LSDM7 cells increased almost twofold at day 14 and almost another twofold at day 21 with respect to day 14. At 21 days, spindle-shaped fibroblasts were observed. At 28 days, all three cultures showed highly filamentous cells with a striking central nucleus. They formed a network, losing contact inhibition and becoming morphologically homogeneous. After 28 days the cells resembled myofibroblasts (Figure 3).

### 3.3. Comparison of B-LSDM7 with Human Lung Fibroblast Tissue from IPF Patient

Cryopreserved ampules of Diseased Human Lung Fibroblasts, Idiopathic Pulmonary Fibrosis (DHLF-IPF) containing ≥ 500,000 cells (Catalog #: CC-7231) were purchased from LONZA. These human lung fibroblasts (HLFs) from a donor with idiopathic pulmonary fibrosis at P3 were expanded for 28 days and used for experiments from P6 to P10. The HLFs were expanded until they formed colonies (about 2 weeks). Part was used for experiments and part was frozen. Supplemented FBM was used for the cell culture. Cultured HLFs were observed by inverted light microscope. The HLFs were partly phenotype. At each passage, they were counted by trypan blue exclusion in a Burker chamber. As shown in Figure 3, the number of HLFs increased almost fourfold at day 14 then at least twofold at 28 days. At 21 days, spindle-shaped fibroblasts began to grow. At 28 days, cultures showed typical features of fibroblasts. Furthermore, the cell culture formed organizing traps.

### 3.4. Tgf-β Administration and Scratch Test

**Figure 4:**
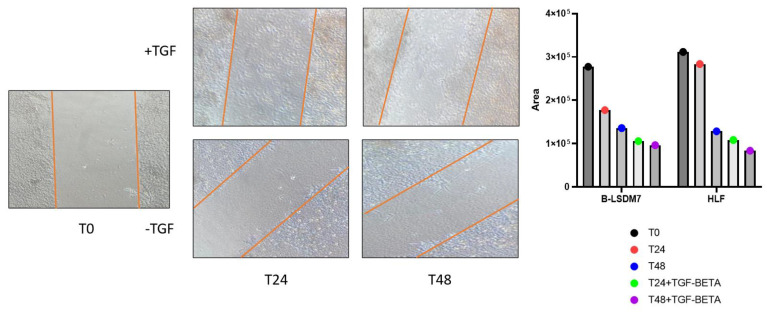
The inverted microscope were used for photo at time 0, 24 and 48 h. At any timepoint, the number of cells encompassed by the lesion area was used as a parameter to define the rate of growth conditions among the experimental settings assessed. Images of cells filling the gaps were acquired 0, 24 and 48 h after generating the lesion.

The scratch test revealed that the B-LSDM7 cell line tended to be more invasive than HLF. Administration of TGF-β induced a profibrotic phenotype in both cell lines; however, slight differences were observed between the two at 24 and 48 h. After 24 h, B-LSDM7 was more invasive than HLF. At 48 h, they were equally invasive (Figure 4).

### 3.5. Characterization of Phenotype

**Figure 5:**
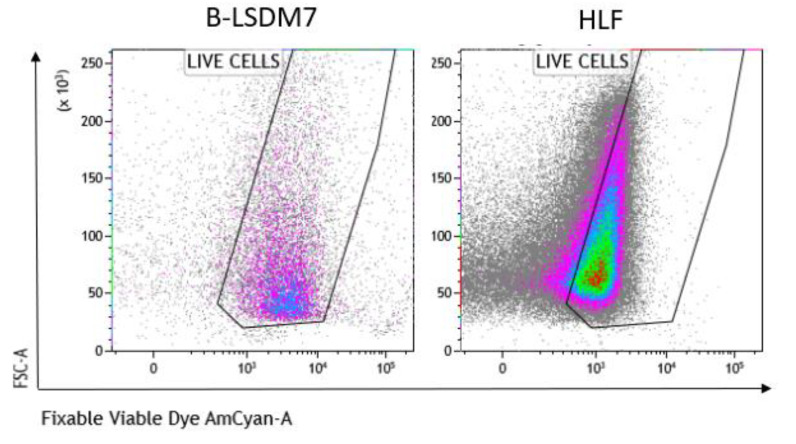
The Flow cytometric analysis of viability in B-LSDM7 and HLF cell lines. Fixable Viable Dye was used for the detection of dead/live cells.

**Figure 6:**
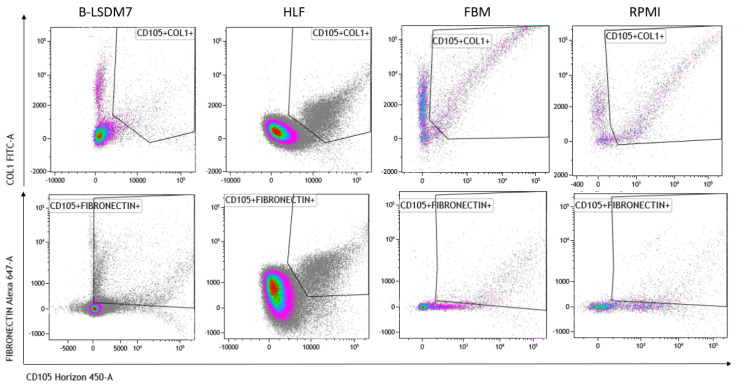
The Flow cytometric analysis of CD105, Fibronectin and Collagen Type 1 in B-LSDM7, HLF, FBM and RPMI cell lines.

**Figure 7:**
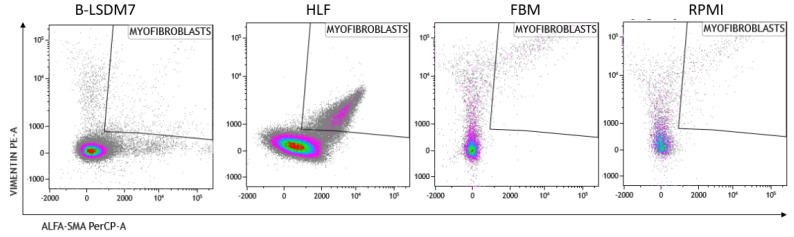
The Flow cytometric analysis of Vimentin and alfa-sma in B-LSDM7, HLF, FBM and RPMI cell lines.

We assessed expression of markers, including type-1 collagen, fibronectin and CD105, which are typical markers of tissue fibroblasts, by flow cytometry. Expression of α-SMA and vimentin signal fibroblast differentiation into myofibroblasts. Cell viability of HLF and B-LSDM7 was also evaluated. Interestingly, the percentage of live cells was similar (69.14% and 64.47%, respectively) (Figure 5). Expression of CD105^+^ Col1^+^ and CD105^+^ Fibronectin^+^ were the same in both cell lines (9.12% in B-LSDM7 and 9.33% in HLF of CD105^+^ Col1^+^ and 7.66% in B-LSDM7 and 88.7% in HLF of CD105^+^ Fibronectin^+^) (Figure 6). Co-expression of α-SMA and vimentin was different in the two cell lines. Myofibroblasts from HLF expressed both markers (14.8%), whereas myofibroblasts from B-LSDM7 only expressed a single marker (1.3% DP; 6.78% of CD105^+^ Vimentin^+^ αSMA^−^ and 8.54 of CD105^+^ Vimentin^− ^αSMA^+^). Both cell lines showed similar percentages of myofibroblasts (Figure 7).

### 3.6. Detection of ROS

**Figure 8:**
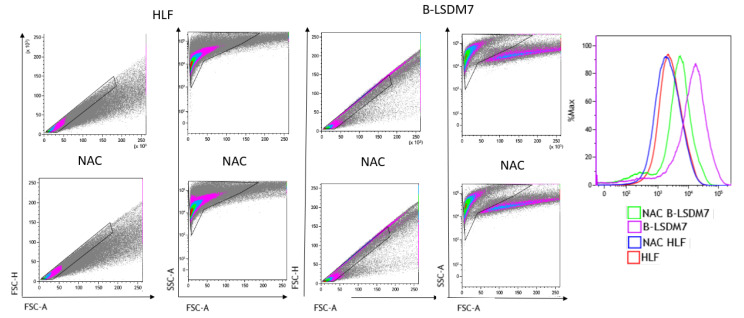
The detection of reactive oxygen species (ROS) through Flow cytometric analysis. The use of N-acetil cysteine was applied as anti-oxidant. The status of oxidation of B-LSDM and HLF were performed through CellROX^®^ Green Flow Cytometry Assay Kits.

**Figure 9:**
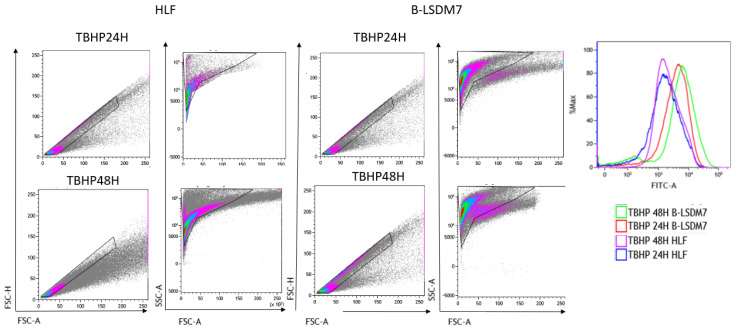
The detection of reactive oxygen species (ROS) through Flow cytometric analysis. The use of TBHP was applied as an oxidant. The status of oxidation of B-LSDM and HLF were performed through CellROX^®^ Green Flow Cytometry Assay Kits.

Flow cytometry assay with CellROX green was used to determine levels of ROS. After gating live singlets based on forward and side scatter, a CellROX^+^ gate was drawn. B-LSDM7 and HLF showed differences in ROS levels measured using CellROX Green and tert-butyl hydroperoxide (TBHP) as positive control. There was a significant dissimilarity between the overall profile of HLF and B-LSDM7. B-LSDM7 showed initial higher levels of ROS than HLF. When N-acetylcysteine (NAC) was administered, a great shift was only observed in B-LSDM7 (Figure 8). After 24 and 48 h of oxidative stress induced by TBHP, HLF showed slightly different levels of ROS, while B-LSDM7 showed high levels of ROS (Figure 9).

**Figure 10:**
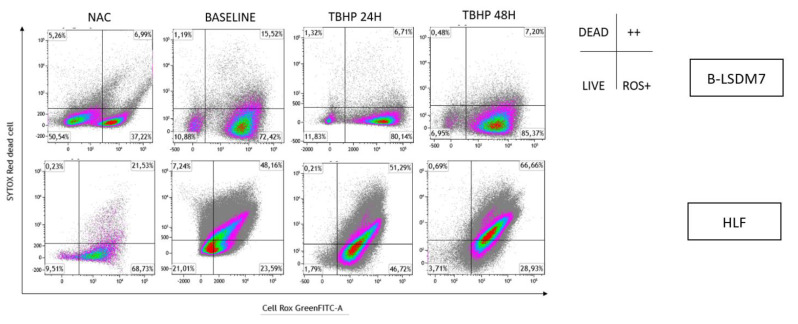
The detection of reactive oxygen species (ROS) and viability of cells following oxidation were performed through Flow cytometric analysis. Sytox Red was used to detect dead cells. The status of oxidation of B-LSDM and HLF were performed through CellROX^®^ Green Flow Cytometry Assay Kits.

Oxidation levels were also integrated with live/dead cell analysis using SYTOX Red staining, as reported in Figure 10. B-LSDM7 and HLF had low levels of dead cells. Many B-LSDM7 cells were oxidized, and their number increased after stimulation with TBHP, though without killing the cells. Concerning HLF, TBHP killed them, and after 48 h, 51% were oxidized and dead.

## 4. Discussion

In this study, a new cultured cell line named B-LSDM7 was isolated from the BAL fluid of an IPF patient. The cell line was expanded in vitro and was phenotypically, morphologically and functionally characterized. This BAL cell line was also compared with commercially available fibroblasts derived from lung tissue of an IPF patient.

From a morphological point of view, we observed that spindle-shaped fibroblasts started to grow at 21 days of culture. At 28 days, cultures showed highly filamentous cells with an evident central nucleus. From the phenotypic point of view, this cell line displays fibroblast/myofibroblast-like features including expression of alpha-SMA, vimentin, collagen type-1 and fibronectin.

The cells are characterized by high amounts of CD105^+^, collagen type-1 and fibronectin. Moreover, co-expression of CD105^+^ Col1 and CD105+fibronectin was the same in HLF and in B-LSDM7. CD105 or endoglin, a cell membrane glycoprotein, is over-expressed on proliferating endothelial cells. It binds several factors of the TGF-beta superfamily and is recognized as a marker of neo-angiogenesis [21]. In the absence of TGF-β1, CD105 shows an anti-apoptotic effect in endothelial cells subject to hypoxic stress, suggesting a protective role of CD105 against pro-apoptotic factors [22]. It has been demonstrated that CD105^+^ fibroblasts permit tumor growth in vivo [23]. In lung fibroblasts, expression of CD105 has been described in BAL-derived fibroblasts of cHP patients [24]. To our knowledge, the first demonstration of expression of this receptor was in HLF and the B-LSDM7 cell line. However, since CD105 positivity is shared with mesenchymal stromal/stem cells [25], we also considered simultaneous expression of collagen type-1 and fibronectin. TGF-β1 increases fibronectin and col1 expression in HLF [26]. The same expression of collagen type 1 and fibronectin was reported in both cell lines. Collagen and fibronectin accumulation is a major feature of pulmonary fibrosis. TGF-β stimulates collagen type 1 and fibronectin production from fibroblasts of normal and fibrotic human lungs [27].

Analysis of vimentin and α-SMA expression in myofibroblasts showed co-expression in HLF but not in B-LSDM7. The ability of fibroblasts to express α-SMA after TGF-β stimulation is well-documented, whereas there is no full documentation of fibroblasts as precursors of myofibroblasts [27]. It remains unclear whether the epithelial–mesenchymal transition first requires a fibroblast phenotype before myofibroblasts can differentiate [28,29]. BAL fibroblasts and tissue-derived fibroblasts may represent an activated state of alveolar fibroblasts, or a difference in phenotype between two distinct populations of fibroblasts originating from two distinct areas of the lung. Activated fibroblasts co-expressing α-smooth muscle actin (SMA) and vimentin are associated with cancer; their extracellular matrix production/remodeling are different from that of resting fibroblasts [30,31]. Vimentin, a cytoskeletal protein in cells of mesenchymal origin, has been associated with increased invasiveness [32]. Over-expression of vimentin promotes increased invasiveness and excessive scarring [32].

Few authors have attempted isolation of fibroblasts from BAL fluid. Quesnel C. et al. tried to culture BAL fibroblasts from ARDS patients and demonstrated that alveolar fibroblasts exhibit a persistent activated phenotype with enhanced migratory and collagen type 1 production capacities [33]. Larson-Casey et al. developed a protocol to evaluate alveolar macrophage-derived TGF-β1 regulation of lung fibroblast differentiation for studying the ability of mouse BAL fluid to alter fibroblast differentiation [34]. Although the literature has no reports of any functional and activity assessments, our study demonstrated a role of oxidative stress. Our functional assay of redox status of B-LSDM7 was performed to highlight the oxidative status of these cells and investigate their behavior under stress. The results showed high expression of ROS in these cells. Oxidative stress invariably promotes extracellular matrix expression in lung diseases directly or through over-production of pro-fibrotic growth factors [35]. It is well known that α-SMA and type-I collagen expression are strongly induced by oxidizing agents. Cultured IPF cells are known to resist peroxide-induced cell death [36]. In our experiment, the cell lines B-LSDM7 and HLF showed low levels of dead cells after oxidative stress was induced. B-LSDM7 cells in particular showed a large number of oxidized cells, which increased after stimulation with TBHP, demonstrating that this phenotype confers resistance to oxidative stress-induced cell death.

The use of FGF-2 was also to establish the growth and development of these cell lines. Conflicting results have been reported in different studies regarding the role of FGF-2. Some authors report that it decreased collagen expression and differentiation of fibroblasts into myofibroblasts [37]. Other papers report that TGF-β1 induces FGF-2 expression and/or release, promoting profibrotic activity [38,39]. Our results sustain the concept that FGF-2 plays a crucial role in the development of typical fibroblasts in the process of fibrosis.

Isolation of cell cultures of human fibroblasts from BAL fluid of IPF patients is a research challenge with several advantages over existing methods. Our protocol makes it possible to obtain fibroblasts from a matrix different from lung tissue, which is of course obtained by an invasive method such as lung biopsy. Lung biopsy is best avoided in patients with lung fibrosis because it can accelerate the disease. BAL is a routine non-invasive method that offers the possibility of having a large sample of patients. However, standardized culture methods are important for a reliable model for testing molecules and eventual therapeutic targets.

The development of new cell lines derived from BAL find different applicability. First of all, they are cost effectiveness, easy to use and also provide a good amount of material. This material also could help bypass ethical concerns associated with the use of animal and human tissue. From a pathogenetic point of view, a pure population of cells is valuable since it provides a consistent sample and reproducible results. Moreover, this cell line can be used in testing drug metabolism and cytotoxicity, antibody production, study of gene function, generation of artificial tissues and synthesis of biological compounds such as therapeutic proteins.

Further studies will be useful to clarify the functions and characteristics of this cell line.

## Data Availability

The data presented in this study are available on request from the corresponding author.

## Availability of Data and Material

The datasets generated during and/or analyzed during the current study are available from the corresponding author on reasonable request.

## Abbreviations

idiopathic pulmonary fibrosis (IPF), bronchoalveolar lavage (BAL), human lung fibroblast from IPF patients (HLF).

## References

[1] Chen X., Guo J., Yu D., Jie B., Zhou Y. (2021). Predictors of Mortality in Progressive Fibrosing Interstitial Lung Diseases. Front. Pharmacol..

[2] Raghu G., Remy-Jardin M., Myers J.L., Richeldi L., Ryerson C.J., Lederer D.J., Behr J., Cottin V., Danoff S.K., Morell F. (2018). Diagnosis of Idiopathic Pulmonary Fibrosis. An Official ATS/ERS/JRS/ALAT Clinical Practice Guideline. Am. J. Respir. Crit. Care Med..

[3] d’Alessandro M., Bergantini L., Cameli P., Fanetti M., Alderighi L., Armati M., Refini R.M., Alonzi V., Sestini P., Bargagli E. (2021). Immunologic responses to antifibrotic treatment in IPF patients. Int. Immunopharmacol..

[4] Cameli P., Refini R.M., Bergantini L., d’Alessandro M., Alonzi V., Magnoni C., Rottoli P., Sestini P., Bargagli E. (2020). Long-Term Follow-Up of Patients With Idiopathic Pulmonary Fibrosis Treated With Pirfenidone or Nintedanib: A Real-Life Comparison Study. Front. Mol. Biosci..

[5] Bargagli E., Refini R.M., d’Alessandro M., Bergantini L., Cameli P., Vantaggiato L., Bini L., Landi C. (2020). Metabolic Dysregulation in Idiopathic Pulmonary Fibrosis. Int. J. Mol. Sci..

[6] Sgalla G., Iovene B., Calvello M., Ori M., Varone F., Richeldi L. (2018). Idiopathic pulmonary fibrosis: Pathogenesis and management. Respir. Res..

[7] Kendall R.T., Feghali-Bostwick C.A. (2014). Fibroblasts in fibrosis: Novel roles and mediators. Front. Pharmacol..

[8] Kim K.K., Sheppard D., Chapman H.A. (2018). TGF-β1 Signaling and Tissue Fibrosis. Cold Spring Harb. Perspect. Biol..

[9] Sisto M., Ribatti D., Lisi S. (2021). Organ Fibrosis and Autoimmunity: The Role of Inflammation in TGFβ-Dependent EMT. Biomolecules.

[10] Sheng L., Zhuang S. (2020). New Insights Into the Role and Mechanism of Partial Epithelial-Mesenchymal Transition in Kidney Fibrosis. Front. Physiol..

[11] D’Urso M., Kurniawan N.A. (2020). Mechanical and Physical Regulation of Fibroblast–Myofibroblast Transition: From Cellular Mechanoresponse to Tissue Pathology. Front. Bioeng. Biotechnol..

[12] Selman M., Pardo A. (2002). Idiopathic pulmonary fibrosis: An epithelial/fibroblastic cross-talk disorder. Respir. Res..

[13] Schildge J., Frank J., Klar B. (2016). The Role of Bronchoalveolar Lavage in the Diagnosis of Idiopathic Pulmonary Fibrosis: An Investigation of the Relevance of the Protein Content. Pneumologie.

[14] Meyer K.C., Raghu G. (2011). Bronchoalveolar lavage for the evaluation of interstitial lung disease: Is it clinically useful?. Eur. Respir. J..

[15] Bergantini L., d’Alessandro M., Cameli P., Otranto A., Finco T., Curatola G., Sestini P., Bargagli E. (2021). Prognostic role of NK cell percentages in bronchoalveolar lavage from patients with different fibrotic interstitial lung diseases. Clin. Immunol..

[16] Bergantini L., d’Alessandro M., Cameli P., Perrone A., Cekorja B., Boncompagni B., Mazzei M.A., Sestini P., Bargagli E. (2021). Integrated approach to bronchoalveolar lavage cytology to distinguish interstitial lung diseases. Eur. J. Intern. Med..

[17] d’Alessandro M., Soccio P., Bergantini L., Cameli P., Scioscia G., Foschino Barbaro M.P., Lacedonia D., Bargagli E. (2021). Extracellular Vesicle Surface Signatures in IPF Patients: A Multiplex Bead-Based Flow Cytometry Approach. Cells.

[18] Tashiro J., Rubio G.A., Limper A.H., Williams K., Elliot S.J., Ninou I., Aidinis V., Tzouvelekis A., Glassberg M.K. (2017). Exploring Animal Models That Resemble Idiopathic Pulmonary Fibrosis. Front. Med..

[19] Maher T.M., Nambiar A.M., Wells A.U. (2022). The role of precision medicine in interstitial lung disease. Eur. Respir. J..

[20] Meyer K.C., Raghu G., Baughman R.P., Brown K.K., Costabel U., du Bois R.M., Drent M., Haslam P.L., Kim D.S., Nagai S. (2012). An Official American Thoracic Society Clinical Practice Guideline: The Clinical Utility of Bronchoalveolar Lavage Cellular Analysis in Interstitial Lung Disease. Am. J. Respir. Crit. Care Med..

[21] Fonsatti E., Sigalotti L., Arslan P., Altomonte M., Maio M. (2003). Emerging role of endoglin (CD105) as a marker of angiogenesis with clinical potential in human malignancies. Curr. Cancer Drug Targets.

[22] Li C., Issa R., Kumar P., Hampson I.N., Lopez-Novoa J.M., Bernabeu C., Kumar S. (2003). CD105 prevents apoptosis in hypoxic endothelial cells. J. Cell Sci..

[23] Hutton C., Heider F., Blanco-Gomez A., Banyard A., Kononov A., Zhang X., Karim S., Paulus-Hock V., Watt D., Steele N. (2021). Single-cell analysis defines a pancreatic fibroblast lineage that supports anti-tumor immunity. Cancer Cell.

[24] Giannoni P., Grosso M., Fugazza G., Nizzari M., Capra M.C., Bianchi R., Fiocca R., Salvi S., Montecucco F., Bertolotto M. (2021). Establishment and Characterization of a Novel Fibroblastic Cell Line (SCI13D) Derived from the Broncho-Alveolar Lavage of a Patient with Fibrotic Hypersensitivity Pneumonitis. Biomedicines.

[25] Denu R.A., Nemcek S., Bloom D.D., Goodrich A.D., Kim J., Mosher D.F., Hematti P. (2016). Fibroblasts and Mesenchymal Stromal/Stem Cells Are Phenotypically Indistinguishable. Acta Haematol..

[26] Bradbury P., Nader C.P., Cidem A., Rutting S., Sylvester D., He P., Rezcallah M.C., O’Neill G.M., Ammit A.J. (2021). Tropomyosin 2.1 collaborates with fibronectin to promote TGF-β1-induced contraction of human lung fibroblasts. Respir. Res..

[27] Raghu G., Masta S., Meyers D., Narayanan A.S. (1989). Collagen Synthesis by Normal and Fibrotic Human Lung Fibroblasts and the Effect of Transforming Growth Factor-β. Am. Rev. Respir. Dis..

[28] Willis B.C., Liebler J.M., Luby-Phelps K., Nicholson A.G., Crandall E.D., du Bois R.M., Borok Z. (2005). Induction of epithelial-mesenchymal transition in alveolar epithelial cells by transforming growth factor-beta1: Potential role in idiopathic pulmonary fibrosis. Am. J. Pathol..

[29] Phan S.H. (2012). Genesis of the myofibroblast in lung injury and fibrosis. Proc. Am. Thorac. Soc..

[30] Nomura S. (2019). Identification, Friend or Foe: Vimentin and α-Smooth Muscle Actin in Cancer-Associated Fibroblasts. Ann. Surg. Oncol..

[31] Origin of Myofibroblasts in Lung Fibrosis | SpringerLink. https://link.springer.com/article/10.1007/s43152-020-00022.

[32] dos Santos G., Rogel M.R., Baker M.A., Troken J.R., Urich D., Morales-Nebreda L., Sennello J.A., Kutuzov M.A., Sitikov A., Davis J.M. (2015). Vimentin regulates activation of the NLRP3 inflammasome. Nat. Commun..

[33] Quesnel C., Nardelli L., Piednoir P., Leçon V., Marchal-Somme J., Lasocki S., Bouadma L., Philip I., Soler P., Crestani B. (2010). Alveolar fibroblasts in acute lung injury: Biological behaviour and clinical relevance. Eur. Respir. J..

[34] Larson-Casey J.L., Carter A.B. (2016). Assay to evaluate BAL Fluid regulation of Fibroblast α-SMA Expression. Bio-Protocal.

[35] Watson W.H., Ritzenthaler J.D., Roman J. (2016). Lung extracellular matrix and redox regulation. Redox Biol..

[36] Bocchino M., Agnese S., Fagone E., Svegliati S., Grieco D., Vancheri C., Gabrielli A., Sanduzzi A., Avvedimento E.V. (2010). Reactive Oxygen Species Are Required for Maintenance and Differentiation of Primary Lung Fibroblasts in Idiopathic Pulmonary Fibrosis. PLoS ONE.

[37] Guzy R.D., Stoilov I., Elton T.J., Mecham R.P., Ornitz D.M. (2015). Fibroblast growth factor 2 is required for epithelial recovery, but not for pulmonary fibrosis, in response to bleomycin. Am. J. Respir. Cell Mol. Biol..

[38] Koo H.Y., El-Baz L.M., House S., Cilvik S.N., Dorry S.J., Shoukry N.M., Salem M.L., Hafez H.S., Dulin N.O., Ornitz D.M. (2018). Fibroblast growth factor 2 decreases bleomycin-induced pulmonary fibrosis and inhibits fibroblast collagen production and myofibroblast differentiation. J. Pathol..

[39] Xiao L., Du Y., Shen Y., He Y., Zhao H., Li Z. (2012). TGF-beta 1 induced fibroblast proliferation is mediated by the FGF-2/ERK pathway. Front. Biosci..

